# Vis Medicatrix Naturæ

**Published:** 1909-12

**Authors:** G. Munro Smith


### "VIS MEDICATRIX NATURÆ."

Inaugural Address at the Annual Meeting of the Bath and Bristol Branch of the British Medical Association, May 26th, 1909.

BY

G. MUNRO SMITH, *President.*

---

I HAVE chosen for my theme "the healing powers of Nature," the *vis medicatrix naturæ.* This phrase was first used, as far as I can discover, by Cullen, who thought that some secret force, which was antagonistic to disease, resided in the animal body. He looked upon most of the symptoms of an illness as the result of a reaction against the causes of disease. Fever, for example, he considered a reparative process, and gout, according to him, was an atony of the digestive organs, against which a reparative effort in the form of a joint inflammation was periodically set up.[1]

This theory of Cullen's, in spite of its obvious errors, has an element of truth in it curiously in accord with some of the explanations of modern physiology and pathology.

In a brilliant and suggestive address given before the British Medical Association at Cambridge, in August, 1880,[2] Sir James Paget urged the importance of a catholic spirit in the study of pathology, and especially laid stress on the lessons to be learnt from the diseases of plants, and Nature's attempts to heal them. He mentioned the fact, for instance (which we have most of us

[1] Park's *History of Medicine,* 1899, p. 198.

[2] *Brit. M. J.,* 1880, ii, 611, 649.

noticed), that if the branch of a tree is broken off (or nearly off) in the summer, the leaves remain on this branch through the autumn and winter, whilst the rest of the tree becomes denuded of leaves. The branch has lost its vitality, and has not the power to cast off or *slough* off its leaves. Here we have a good example of one of Nature's means of healing wounds and getting rid of dead tissue, that is sloughing, with the formation of granulations.

He further calls attention to the overgrowths or hypertrophies produced by the pricks of insects, etc. (*e.g.* gall-nuts), as a method of protection ; the healing of the trunk after the amputation of boughs by the skinning over of the under and upper layers of the bark, etc. Both animal and plant life are, indeed, full of instances of this *vis medicatrix naturæ.* The subject is important to all of us, for there can be no doubt we cannot do battle with disease or accident successfully unless we study the processes by which Nature cures. Moreover, these phenomena are so marvellous, that a man must be very dull, or very "superior" indeed in his own estimation, if he sees nothing in them worthy of his admiration. "Surely," says Sir James Paget, "in our familiarity with the processes of repair we overlook their wonder ; the wonder that there should be in anything an inherent power of repairing the consequences of accident."

I am fully aware of the difficulties of my task. The more one thinks of these mysterious attempts at protection, immunity, healing and reparation which go on in the body the more complicated the subject grows. I hope, however, to avoid controversial questions, and confine myself (1) to the attitude taken by medical men towards Nature's healing powers, (2) to some instances in which these powers are well shown, and (3) to a few thoughts thereon which seem to suggest themselves.

I said that there were difficulties in the way. These are chiefly connected with the abstruse nature of the processes involved, for it is evident that a consideration of the *vis medicatrix naturæ* touches on many philosophical problems, and it would be easy, unless one carefully picked one's way, to fall into the quagmires of theological discussion, where one might flounder helplessly. I shall not waste your time by defining what I mean by the word "nature." Everyone has, I trust, read *Nicholas Nickleby*, and remembers Mr. Squeers. "'She 's a rum' un, is Natur'," said Mr. Squeers. "She is a holy thing, sir," remarked Snawley. "I believe you," added Mr. Squeers with a moral sigh, "I should like to know how we should ever get on without her. Natur'," said Mr. Squeers solemnly, "is more easier conceived than described. Oh! what a blessed thing, sir, to be in a state of Natur'!" [1]

Let us, for once, agree with Mr. Squeers that Nature is a "rum' un," and that she is "more easier conceived than described," without further definition.

It is certain, at all events, that in animals and plants there take place, constantly, certain processes which are antagonistic to disease, which tend to repair losses of tissue, and heal, or attempt to heal, injuries.

Every medical man must work in alliance with these processes or must thwart them. He may fight disease in the old-fashioned way, "armed with a mortar and a pestle," or equipped with a top hat and a look of unnatural wisdom, or with every available help from modern science; but in any case his success will depend on his attitude to the *vis medicatrix naturæ*. The neglect of Nature's methods, or the active opposition to them, has brought doctors in all ages under the lash of ridicule. There has always prevailed some idea that the medical man, instead of helping Nature on the lines she indicates, has opposed her by his drugs, his operative interference, and so forth. The unfortunate patient often feels this, and even argues about it. When Rabelais was in his last illness, and a consultation was held at his bedside, "Pray," said he, "pray, let me die a natural death!" And those who have read Moore's *Life of Byron* will remember the painful scene at Missolonghi, where Byron, weakened by insufficient food, and in a state of nervous irritability, pleads with his medical men that he should not be bled, quoting Dr. Reid's statement that less slaughter was caused by the lance than the lancet. But there were two to one—two doctors to one patient—and he was at length, weary with the discussion, forced to consent,

[1] *Nicholas Nickleby*, chap. xlv.

and was bled, first to twenty ounces ; and this was repeated next day, not because he was better, but because he was worse ; and not content with this heroic treatment, the poor poet was blistered on both legs.[1]

We do not bleed much now, or even blister, but drugs are almost as much to the fore as ever they were; and, indeed, most patients insist on having something to take three times a day after meals ; they have become so accustomed to this, that if it is not ordered they think they are sadly neglected. Yet this kind of treatment, although often necessary as a piece of diplomacy, is in many cases not done with any definite plan of bringing about a cure by any physiological process.

Montaigne[2] declared that he never took medicine. He remarks, " And answer such as urge me to take Physicke that at least they will tarie till such times as I have recovered my health and strength againe; that then I may the better be enabled to endure the violence and hazard of their potions. I let Nature worke, and presuppose unto my selfe that she hath provided her selfe both of teeth and clawes to defend her selfe from such assaults as shall beset her." These teeth and claws constitute the weapons of the *vis medicatrix naturæ.*

Montaigne says in another place,[3] still speaking of doctors: " I never meddled with the bitterness of their prescriptions. I send for them if I am sicke, if they may conveniently be found; and love to be entertained of them, rewarding them as other men doe." You will be pleased to hear, however, that he owns to " having knowne diverse honest men among them : it is not them I blame, but their arte."

Others were not so lenient as Montaigne. It was not very uncommon for an unsuccessful physician to be put to death ; and at one time it was gravely debated whether, if a physician were called to a case and found it hopeless, he should not either run away, to escape possible slaughter, or call in a chirurgeon, on whom he could fix the blame in case the patient died—a plan still

[1] Moore's *Life of Byron*, p. 635,

[2] Montaigne's *Essays*, book i, chap. xxii (translated by John Florio).

[3] *Ibid.*, book ii, chap. xxxvii.

sometimes adopted with success. In Withington's *History of Medicine* it is stated that Austragild, wife of Guntram, King of the Burgundians, before her death, begged her husband to execute her two physicians on her tomb. The King did so; but Bishop Gregory's comment on the proceeding is "that many considered that the deed was not without sin."

In the same entertaining book[1] is an abstract from a mediæval work entitled *De adventu Medici*, which although not very cognate to my subject, yet is so interesting as showing how like medical men are in all ages that I quote it:—

"When called to a patient commend yourself to God and to the angel who guided Tobias. On the way learn as much as possible from the messenger, so that if you discover nothing from the patient's pulse or water, you may still astonish him and gain his confidence by your knowledge of the case. On arrival ask the friends whether the patient has confessed, for if you bid him do so after the examination it will frighten him. Then sit down, take a drink, and praise the beauty of the country and the house, if they deserve it, or extol the liberality of the family. Next proceed to feel his pulse, remembering that it may be affected by your arrival, or the patient being a miser, by his thinking of the fee. . . . Tell the patient that you will cure him, with God's help, but inform the friends that the case is a most serious one," etc.

These excellent rules might serve, with very little alteration, for the conduct of a case to-day. As stated, the idea that doctors not unfrequently shortened patient's lives by their treatment, and that if left to Nature the cases would do well enough, has always been common. In an ancient book called *Wilson's Arte of Rhetorique*, published in 1560, there is the following: "When an unlearned Phisitian (as England lacketh none such) had come to Pausanias, a noble Gentleman, and asked him if he were not troubled much with sicknesse, 'No, Sir' (quoth he), 'I am not troubled at all, I thanke God, because I use not thy counsaile.' 'Why do ye accuse me' (quoth the Phisitian) 'that never tried me?' 'Marie' (quoth Pausanias), 'if I had once tried thee,

[1] Withington's *History of Medicine*, 1894.

I should never have accused thee, for then had I been dead and in my grave many daies agone.' "

I hope these quotations will not lead anyone to think that I underrate the proper use of drugs or other remedies and advocate a system of *laissez-faire.*

In fact, I agree to a great extent with some remarks made by my father in 1847, in reference to a controversy on this subject in the *British and Foreign Medical Review.*[1]

He says: "The author is deeply convinced that investigation will only lead to a more reverential and implicit trustfulness, and to a more complete recognition of the value of the science and practice of medicine, as it has descended to us, enriched by the researches of ages. Fully is he convinced that, making the proper allowance for an alloy of human error, it is a vast and magnificent commentary on the book of Nature."

The discussion that my father refers to is so important in relation to my subject, that I will say a few words about it.

In the *British and Foreign Medical Review* for January, 1846, appeared an excellent criticism by the then editor, Dr. Forbes, on several books on Homœopathy. This review is characterised by a remarkable fairness and judicial tone.

He does not condemn Hahnemann off-hand, as is so often done, as a mere ignoramus. He gives him credit for his erudition and investigations, although he is quite alive to the *a priori* absurdity of some of his ideas. To take one instance of many. Hahnemann, as everybody knows, came to the conclusion that extremely dilute solutions of a drug have a powerful effect if the dilution is made under certain conditions. He says, for example, that the decillionth of a grain of oyster-shell will take forty days to produce its full effects on the patient. Now a decillion is the tenth power of a million, and is written by 1 followed by 60 ciphers. Such a dilution would be something like dissolving a teaspoonful of salt in Lake Windermere. But Dr. Forbes is impressed with the fact that according to fairly reliable statistics patients at that time recovered from typhoid,

[1] "A Few Remarks on the Expectant Treatment of Disease." By ΑΚΕΣΤΗΣ. John Churchill. 1847.

small-pox or measles as well under this treatment as under the orthodox bleeding, purging, antimony, etc., and he asks: Do patients get well because of the treatment or in spite of the treatment?

He emphasises this by an incident which happened in his own practice. A little girl had been under his care, and he asked her on his visit how she was. "Oh," said she, "I 'm very much better, sir." "And how long have you been so much better?" "Since Friday, sir." "Ha! What happened on Friday?" "Please, sir, my medicine was done!"

He sums up by drawing certain inferences, viz.:—

(1) A large proportion of patients are cured by Nature, not by the physician.

(2) In a lesser, but still not a small, proportion the disease is cured by Nature in spite of the physician.

(3) That consequently in a considerable proportion of diseases it would be as well or better with patients if drugs were abandoned.

The general trend of this paper was backed up by Dr. Andrew Combe (in April, 1846), who said: "Co-operate with Nature instead of taking her by storm." In fact, he lays stress on the *vis medicatrix naturæ*, and advocates the general care of cases by nursing and so forth instead of drugging. He was before his time, and the orthodox fell upon him in the shape of Dr. Addington Symonds, who claimed (October, 1846) that as man has succeeded in mastering so many powers of Nature, he might, by analogy, be supposed to acquire some dominion over the *vis vitiatrix* and the *vis necatrix*, which have to be dealt with as well as the *vis medicatrix*. He says: "Though she (Nature) knits up wounds with her adhesive inflammation, by the very same method she glues the intestines into fatal entanglements, shackles the heart and closes up the windpipe."[1]

Let us for a moment consider these arguments of Dr. Symonds in the light of present knowledge.

As regards the *vis vitiatrix*, it would be difficult to prove

[1] *British and Foreign Medical Review*, p. 559. October, 1846.

that there was such a force. In the progress of evolution it is essential that some such force as the *vis medicatrix* should come into existence for the sake of the organism. But there is no reason for the existence of any thing (call it force or principle or what you will) which would tend to the detriment of the organism.

The *vis necatrix* or tendency to death, became necessary to ensure variation and development, it is true, but Dr. Symonds certainly was not thinking of this in 1846. As to his argument that the same process which heals a wound also causes fatal peritonitis, the adhesive inflammation of peritonitis is set up probably as a protective process, to keep the inflamed intestines from movement ; and even if this is not allowed as an argument, yet it is obvious that the leucocytosis which accompanies the plastic inflammation is entirely in the direction of healing. Moreover, when you begin to speak of diseases due to germs and parasites, one must remember that Nature looks after the humblest of her products, and provides means for its protection and propagation, just as she provides for her crowning work—man. A good instance of this may be given from the natural history of the filaria sanguinis hominis. Sir Patrick Manson has shown that this parasite is found in the blood under the skin, that is, in the superficial circulation, only, or chiefly at night. Why is this ? Probably because it is important for the filaria to be carried from host to host, from one pasture to another, and it is carried from the patient's blood by the nightly visits of mosquitoes. This determination of the parasite to the superficial blood-vessels at night is probably, says Sir Patrick, an "adaptation of the habits of the filaria to the nocturnal habits of the mosquitoes."

The above discussion seems to me all in favour of giving Nature a tolerably free hand, and it is interesting to note that according to recent statistics one of the diseases mentioned, namely typhoid fever, when treated by what may be called the expectant method, shows 80 to 90 per cent. recoveries.[1]

I will now briefly mention one or two of Nature's methods

[1] "Principles of Vaccine Therapy," *Lancet*, 1907, ii, 423.

of cure. One of the most interesting is the formation round the offending body (whether it be necrosed tissue, germs, tumours or extraneous substances) of a case or capsule, so that the cause of irritation is covered up and kept from injuring the surrounding tissues, or cut off from its hlood supply and starved, so that it subsequently undergoes degeneration and perhaps absorption.

The encasement of dead bone, the isolation and consequent caseation and calcification of tubercular deposits, the formation of tissue round foreign bodies, etc., will occur as examples of this.

A good instance of this is seen in the fibrous deposit sometimes formed round cancerous emboli.[1]

It is by a somewhat similar process that Nature treats cancer. The amount of actual carcinomatous material in a slowly-forming scirrhus of the breast is very small compared with the fibrous tissue round it and in its interstices, which does not belong intrinsically to the disease, but is doing what it can to isolate and check it. That attempts are constantly made in the body, however unsuccessful they may be, to cure cancer has been recognised by various observers. For example, Mr. Pearce Gould has recorded a case of natural recovery from extensive recurrent carcinoma, in the first place mammary,[2] and Sir Watson Cheyne another spontaneous disappearance of recurrent carcinoma.[3]

Mr. W. Sampson Handley (lecture delivered before the College of Surgeons of England on " The Natural Cure of Cancer "[4]) has pointed out how every mass of cancer cells tends to undergo degenerate changes, beginning in the centre and extending to the periphery. This is a very old observation, and is seen in the umbilication or pitting of cancerous nodules in the liver and elsewhere, the centre of the mass becoming necrosed and shrinking. This occurs partly because the central cells do not get so much nourishment as the peripheral cells, but also because every cancer cell has a certain duration of life, and the first formed

[1] *See* Schmidt's picture in *Essays on the Position of Abdominal Hysterectomy in London*. By J. Bland-Sutton. 1909.

[2] *Tr. Clin. Soc. Lond.*, 1897, xxx, 205.

[3] Royal Society of Medicine, May 8th, 1908. [4] *Brit. M. J.*, 1909, i, 582.

naturally die first. But another process is at work, namely the formation of fibrous tissue; and according to Mr. Sampson Handley's "permeation" theory, as the malignant disease extends in a radiating manner from its original birthplace it is followed up by fibrotic changes, which block the lymphatics and obliterate them. At the growing edge of cancer connective tissue cells are formed, and appear to nourish the growth; but when the fibrous tissue is fully formed it acts as an effectual barrier to its onward progress.

If this fibrous tissue could only be formed quickly enough—if it could precede instead of follow the growth—spontaneous cure would be much more frequent. If the enemy, to use a metaphor, could be not only pursued but headed off, it might be entrapped, encapsuled and killed. Here Nature is in conflict with disease, but she is too slow. Time is of no account with her. The cancer will be killed in time, but the patient is killed whilst the cure is in progress. The process which in this case she does not carry out quickly or thoroughly enough is carried out in other instances too thoroughly, and we get fibrous nodules or tumours round some diseased part or foreign body, just as we get the curative process of sloughing, with granulation tissue and formation of pus carried too far and the patient killed by exhaustion. This leads to the mention of two laws in connection with the *vis medicatrix naturæ*. One is that the reaction against disease is apt to be in excess of the needs; the other is that Nature regards the part more than the individual.

There are other means used in the attempt to cure cancer: there is the natural antagonism of the blood, so that epithelial cells cannot well live in the blood stream; and it is possible that the ferments which exist in all living cells may exert an influence to bring about amelioration. It can be shown that if any organ be kept at the right temperature after it is dead it becomes soft, and digests itself. This autolysis by the intra-cellular ferments is one of Nature's most potent means, probably, for dissolving useless or harmful products, such as infarcts, syphilitic growths, and occasionally malignant tumours. The rapid liquefaction of grey hepatization in pneumonia is probably due to the same means.[1]

Nature can not only destroy diseased parts, but can to a great extent re-form and renew lost tissue.

In April, 1908, I saw a small boy, aged ten, who had suffered from a whitlow on the thumb. The nail was loosened from its bed, and a discharging sinus led down to necrosed bone. I operated on him on April 3rd, 1908, and found the terminal phalanx quite loose and completely necrosed. I took it away, removed the nail, and cut away the diseased skin; in fact, I did what I should describe as amputation at the joint, and I told the parents that he would not have any end joint to his thumb. In January of this year (1909) I saw the boy again. To my great astonishment he had a well-shaped nail on his thumb, and quite a respectable terminal phalanx, with a perfect phalangeal joint. I had a skiagram taken, which I will show you. I have never seen a better example of the *vis medicatrix naturæ*. What happened, no doubt, was that the epiphysis was left, and from this Nature is fashioning a most presentable phalanx.

How far lost parts can be reproduced in man is a problem. It is possible that in early stages of intra-uterine development this may occur; but there is no evidence of a new limb growing that is at all trustworthy. But in the lower animals such phenomena are common. Many of us have seen a lizard cast its tail, and the lost member is quickly reproduced, sometimes growing double. A garden worm cut into two becomes two worms, one part growing a head, the other a tail. Here we have an instance of the rule that Nature considers the lesion but not always the individual, for the tail end sometimes grows a tail instead of a head, leading, of course, to the death of the animal from inanition, rectal feeding being unknown amongst worms.

Star-fish will grow fresh rays to replace amputated ones; crabs cast their claws, and speedily grow fresh ones; the salamander will grow new legs; and the snail will grow such a complicated organ as the eye, if it is cut off, not once but many

[1] Dr. Flexner, *Univ. Penna. M. Bull.*, 1903–04, xvi, 185.

times. The rule seems to be that if a limb or any other part of an animal necessary to its existence is subject to frequent accidental mutilation, Nature in progress of time endows the species with the power of renewing the lost part. The age of the animal makes a great difference ; the younger it is, the greater is the recuperative force.

Sir James Paget has formulated a law on this subject, viz. "that the reparative power bears an inverse proportion to the amount of power consumed in the development and growth of the individual, and in its maintenance in the perfect state."[1]

According to this, we should, of course, expect man to have a less perfect faculty for regeneration of parts than most other animals.

Perhaps, however, the most wonderful instances of the power in our tissues which is constantly fighting against disease and death is to be found in the phenomena of immunity. John Hunter showed by experiment that fresh blood destroyed putrefaction to some extent ; in fact, he and his friend Edward Jenner may be considered the pioneers in this subject. Sir James Paget, in his lecture on dissection wounds, says the "fact of an acquired immunity seems certain." But it was not until bacteriology became a science, and Metchnikoff and Nuttall investigated the action of the leucocytes and blood serum on germs, that the modern study of this subject began to be so important a factor in medical science. Many theories have been promulgated to account for the facts ; but the practical point is this, that the healthy organism has the power of producing substances which are antagonistic to the life of bacteria, foreign cells and cell products, sometimes destroying the cells or germs, sometimes attacking the poisonous substances they secrete.[2] "The essential feature of vaccine therapy," says Sir A. Wright, "is the scientific exploitation for therapeutic uses of the protective machinery with which the organism is equipped."[3]

Under the stimulus of the invading microbes and their

[1] Lectures on Surgical Pathology, 1876.

[2] See the Huxley Lecture for 1902, by Dr. Welch, *Johns Hopkins Hosp. Bull.*, 1902, xiii, 285.

[3] *Lancet*, 1907, ii, 423.

products, not only does "every adult leucocyte become phagocytic," become converted from a peaceful citizen to a kind of "territorial," but these minute warriors are rapidly mobilised, silently, without tap of drum, like those spectral hosts that in Longfellow's poem fought before the walls of Prague. Moreover, substances are formed—the now well-recognised anti-bodies—which have, many of them at least, a specific relation to the body which leads to their formation.

Inasmuch as injurious substances—germs and their products—are constantly introduced into our body, this battle must be an almost every-day occurrence. Probably, as suggested by Dr. Sim Wallace,[1] the microbes of disease frequently find their way into our mouths and into our systems, and by inducing the formation of anti-bodies, lead to a gradually-formed immunity, whereby we—medical men especially—perhaps escape many infectious complaints.

To return for a moment to our simile of the internecine battle between our protective and healing organisations on the one hand and the disease on the other, the mobilisation means the flooding of the affected part with plasma and leucocytes, and the inflammatory process, with its redness, swelling and heat, is part of the *vis medicatrix naturæ.*

The patient's whole temperature is frequently raised, it is true, but this very rise of temperature is probably protective. Rolly and Meltzer[2] have found, experimentally, that phagocytic action is most potent, not at the normal body temperature, but at 104° F., and the production of antitoxins, etc., is more active when the temperature is raised. This would indicate that the mere reduction of fever is not always to be attempted.

This reaction against noxious material by the formation of specific antidotes, and by the machinery of a well-drilled army of leucocytes, is difficult to explain. Probably the whole process is an exaggeration of normal physiological functions. Ehrlich, as is well known, has formulated what is called a side-chain theory, which is, at all events, useful as a working hypothesis.[3]

[1] *Med. Press and Circ.*, April 28th, 1909. [2] *Brit. M. J.*, 1909, i, 1254.

[3] *Die Ehrlichsche Seitenketenttheorie, erläutert und bildlich dargestellt.* von Dr. P. Schatiloff.

Besides the central principal groups, of which cell protoplasm is built up, there are, says Ehrlich, a number of lateral groups or receptors, whose normal function it is to pass on nutriment to the central protoplasmic group. They have the power, however, of also combining with various toxins, and when the invading poison irritates the cell these receptors are thrown off in large numbers, unite with the toxins and neutralise them, and so prevent these toxins from entering into harmful combination with the chief chemical groups of the cell protoplasm ("Leislungskern"). These receptors constitute the anti-bodies, and illustrate the law of regenerative over-compensation formulated by Weigert.[1]

Here, however, we must again pause to remind ourselves that we are "not the only pebbles on the beach." If we have these marvellous curative and protective mechanisms provided for us, what about the poor microbes? They have a life to lead, and complex functions of assimilation and secretion of their own, which are, after all, "a reflex in little" of our own existences. Well, Nature has provided for them also, and it seems probable that the anti-bodies formed in our blood for the destruction of the invading *materies morbi* cause the formation of bodies (perhaps by the over-production of microbial receptors) which in their turn tend to protect the invading hosts by reacting with and destroying the poisons which we produce for their destruction.

Nature, we find, distributes her rewards and penalties equally on man and the smallest organisms, just as, in the words of Keble, the sunshine "lightens worlds or wakes an insect's mirth."

And in studying the laws of Nature we must include amongst them those forces which are classed under the convenient heading of the *vis medicatrix naturæ.* Whatever the future of the medical profession may be, it seems to be almost certain that the physiological processes and chemical reactions which I have so inadequately touched upon to-night will have to be studied deeply. In other words, physiology and pathology, in their widest sense, will be recognised more and more as the groundwork of medicine.

[1] *Studies in Immunity.* By Robert Muir. 1909.

In considering the curriculum of the future medical student, one cannot but be appalled at the amount of lectures on various subjects that the poor wretch will have to submit to, so that by the time he takes out his course of demonstrations at a lunatic asylum he will, one would think, be qualified as an inmate of that institution. He will, at the end of his course, be like the madman in *Punch*, who thought he was a poached egg, and went about looking for a piece of toast to sit upon. If one may venture to make a prophecy it is this: that before many years are over medical study will have to be simplified, and that the first two or three years will be devoted to chemistry, anatomy, physiology and pathology, and the rest will not be at a medical school at all, but in the wards of a hospital, the student occasionally, of course, following his cases into the *post-mortem* room.

Thinking over what I have written, I fear the general impression may be that I have scoffed at holy things, such as drugs and physicians, and who am I that I dare scoff? But I have only intended to express my disbelief in empirical drugging when it has no reason to be trusted from experience, or is not founded upon some physiological principle. I own that many of our most noted remedies are empirical. Until a thing has been tried on man or animals one cannot say much. But the whole history of medicine shows how great faith one generation has had in a mode of treatment that the next generation utterly disbelieves in. Priscian[1] says, for example, that "asses' dung dried and used as a dentifrice will immediately cure toothache." But I have not tried it.

In an old book called *Anglo-Saxon Leechdoms*[2] there is a simple remedy for lunacy, as follows: "In case a man be a lunatic, take skin of a meer-swine or porpoise, work it into a whip; swinge the man therewith; soon he will be well. Amen."[3] I should like to try this on some of my patients, but I dare not. But many remedies have less to recommend them. No doubt they had their day, as every new drug has. Special lines of treat-

[1] Withington's *Medical History*, p. 176.

[2] Master of the Rolls Series. [3] *Ibid.*, p. 181.

ment "have their day, and cease to be." Sometimes the patient "ceases to be" also.

In conclusion, I cannot help feeling that in studying the various processes that go on for our protection and recovery from disease and accident in the "holy secrets of our microcosm," there is something which appeals very powerfully to the imagination. To the man who takes a hopeful view of things the *vis medicatrix naturæ* is a great beneficent force, or series of forces. To the pessimist the whole thing merely emphasises the struggle between good and evil. To the latter I would quote the words of Kant, the great German thinker: "Der mensch will eintracht, aber die Natur weiss besser was für seine Guttang gut ist; sie will zwietracht." "Man would prefer harmony, but Nature knows better what is good for her race; she insists on a discord."[1]

### CASES OF PNEUMONIA.[2]

[1] Quoted by Dr. Baudaline, *Med. Rec.*, 1903, lxiv, 81.

[2] Read at the meeting of the Bath and Bristol Branch of the British Medical Association, held at Bristol, October 27th, 1909.

